# Breastfeeding selfies as relational practice: becoming a maternal subject in the digital age: a single case study

**DOI:** 10.1186/s13006-019-0218-9

**Published:** 2019-06-11

**Authors:** Sharon Tugwell

**Affiliations:** Department for Psychosocial Studies, Birkbeck College, University of London, London, UK

**Keywords:** Breastfeeding selfies, Breastfeeding, Maternal subjectivity, Recognition, Online support groups

## Abstract

**Background:**

In 2015, the popular online parenting forum, *Netmums*, named breastfeeding selfies as the number one parenting trend in the UK for that year. Public reaction to the rise in popularity of this practice is polarised, much like breastfeeding in public. The unspoken rule that breastfeeding should be discreet is challenged by the ostentatious presence of breastfeeding selfies.

**The case study:**

This paper focuses on a detailed case study with a white, working class, single mother of two children who has taken and shared breastfeeding selfies online. The analysis employs psychoanalytic and phenomenological methods in order to consider the interrelation of both the internal processes and external forces at work in the practice of taking and sharing breastfeeding selfies. The focus is on how her practice might function in relation to the development of a maternal subject position and the ways in which any cultural capital associated with breastfeeding is perceived and mobilised. The analysis reveals how the relational dimension of selfie taking and participation in online breastfeeding and mothers’ groups helps develop an embodied sense of cultural capital which has ramifications in the everyday, although not without its own contradictions. Whilst breastfeeding may take up a particular place in contemporary discourses around parenting and ‘good mothering’, the capital it affords women is inherently wrapped up in their subject position and material conditions. Online spaces allow for manoeuvre and the mobilisation of this capital in a way which is precluded in the outside world. The practice of sharing and consuming breastfeeding selfies critically contributes to the actualisation of this capital in an embodied sense.

**Conclusions:**

The key theme which emerges is the crucial need for recognition at both the micro and macro level and how this need for recognition is informed by both psychic and social pressures. The visibility, or self-exposure, associated with selfie sharing contributes to the surety of taking up a maternal subject position, from which the participant was better placed to work through some of the cultural ambivalences she too had internalised toward breastfeeding.

## Background

In both popular and scholarly writing, ‘selfies’ are often positioned as though there is a certain homogeneity to both the practice and the image. Selfies are of course united in the fact that they are a technology-driven phenomenon, reliant on both smart phones with front facing cameras and access to online digital networks. But, of course, there is much more to selfies, as both cultural objects and cultural practices, than the technology that makes them possible. Selfies are a complex and nuanced phenomenon, and the multiple ways in which different *types* of selfies intersect with prevailing cultural discourses, and function as a means of communicating these disruptions, is overlooked when they are, by definition, reduced to the technology which enables them. A meta-narrative of selfies requires an over-simplification in which intersectionality, multiplicity and specificity are denied [1]. In 2015, the popular online parenting forum, *Netmums*, named breastfeeding selfies as the UK’s number one parenting trend of that year. It was during this year that ‘brelfies’ as they’d become known, really hit the news in the UK and the practice could be seen as both popular and widespread among many British mothers [2–4]. Discussion of the phenomenon on both daytime talk shows and online news media revealed a certain polarisation in reactions to breastfeeding selfies, reactions which are on a continuum with wider public debates around breastfeeding in public. With brelfies, it was not so much the content of the image that was the problem but the form, it was the fact that these images were being shared or made public, that was contentious.

The specificity of breastfeeding selfies is denied when we fail to differentiate them from ‘*the* selfie’ more generally and ‘selfie culture’. As cultural objects, selfies are demeaned and devalued; they are rendered substance-less, indicative of a self-obsessed, individualistic and narcissistic culture [5–7]. The devaluation of selfies, and associated refusal to consider the nuanced specificity of breastfeeding selfies, facilitates not only a rejection of the significance or value of this relational practice but also involves pathologising the mother’s desire to take and share her breastfeeding image in the first place [8]. It is worth noting the ways in which the labels of ‘narcissist’ or ‘exhibitionist’ have been applied to particular groups in recent history. As Kristen Dombek [9] points out, the traits and characteristics of the ‘narcissist’ have shifted and will continue to shift ‘according to who’s got the power of diagnosis’. Christopher Lasch’s *The Culture of Narcissism* [10] was perhaps the most influential text when it came to theorizing cultural narcissism [11], in which he observed that narcissistic personality types had become the norm in late modern capitalist American society. The individual had triumphed over the collective through an incessant competitiveness, which was a *fait accomplis* for capitalism, insofar as the value of a human being was synonymous with the value accrued by culturally intelligible symbols of wealth. Although one could argue that Lasch’s analysis was an attack on consumerism, it is important to ask who or what consumerism was believed to be threatening. According to Imogen Tyler [11], Lasch’s analysis required the model of the male, patriarchal family as the benchmark from which contemporary standards were slipping away from. As such, it was not so much the rise of consumerism that was the issue but instead the rise of identity politics and the visibility of individuals who challenged the stability of the patriarchal familial model, for example gay men, lesbian women and single mothers.

This is relevant when considering reactions to breastfeeding selfies, who or what is being threatened by the rise in visibility of breastfeeding through the popularity of breastfeeding selfies? Tyler’s critique of Lasch’s work exposed the patriarchal nostalgic idealization that was in play, it was those who were losing the surety of their social and cultural position that were affected, not those who were just beginning to become visible and assertive in their own right. As such, the question of who and what is legitimised through visibility in society, and conversely who and what remains excluded and unseen, must not be overlooked when considering the cultural reactions to the rise in breastfeeding selfies. Breastfeeding is positioned as an intimate, private act and the audacity of sharing such images reeks of a ‘naked exhibitionism’. These were the words that journalist Angela Epstein used to describe the practice of breastfeeding selfies when the phenomenon was being discussed on ITV’s *Good Morning* show in February 2015. Online news media sites *MailOnline*, *Huffington Post* and *Buzzfeed* subsequently continued the ‘debate’ and the comments in response to these pieces demonstrated the acute polarisation in attitudes toward the practice. A woman breastfeeding in a public space in the ‘real’ world can always defend her position insofar as she is responding to her child’s need to feed. (This, of course, does not make her immune from criticisms related to perceptions of discretion, the suitability of the space for infant feeding, her own appearance and behaviour whilst breastfeeding and so on). However, a woman sharing a breastfeeding selfie online has no such defence. Breastfeeding selfies, by their very nature of being contrived cultural objects, require the desire of the mother to not only take the photograph but then to upload and share it on social media. The backlash against breastfeeding selfies, that they reek of naked exhibitionism for example, is in fact a backlash against the expression of maternal desire. It is not so much the image of a woman breastfeeding, but it is the fact that this same woman not only took the photograph but also had the audacity to display it publicly.

The notion of a clear and rigid distinction between public and private serves an ideological purpose which, following this binary logic, aligns women more closely with the ‘private’ sphere, with all the associations of domestic labour and childcare responsibilities attached to it [12]. Online digital networks and the ordinariness of connecting through social media unsettle this distinction and, in turn, help facilitate and increase women’s presence and visibility in the public domain. Rymarczuk and Derksen suggest that whether individuals like or are uncomfortable with *Facebook* depends upon how attached they are to the distinction between public and private [13]. This is important to hold on to when thinking about negative reactions toward the increased visibility of breastfeeding selfies and the negative reactions toward public breastfeeding more generally. If the boundaries between public and private are being eroded, then there will be a nostalgia for this divide among some people, once again it is important to ask whose position might be threatened by the blurring of such distinctions? Breastfeeding is positioned as a private, intimate, individual act and its circulation and currency in public, social fields (be they online or not) gives rise to a backlash, an urge to put breastfeeding ‘back where it belongs’, out of sight [14]. The increased presence of breastfeeding selfies on social media sites such as *Facebook* and *Instagram* have largely been in response to an ongoing censorship war between the platforms and the women who post breastfeeding selfies online. The rise in the popularity and visibility of breastfeeding selfies in the UK in 2015 was largely due to such an occurrence. In October 2014, a British woman posted a breastfeeding selfie of her and her prematurely born, two-week-old daughter on her *Facebook* page. It was reported, presumably by someone on her ‘friends’ list, and the image was subsequently removed by *Facebook* because ‘it didn’t follow *Facebook* Community Standards on nudity’. The mother then posted the image to a breastfeeding support group on *Facebook* and it quickly went viral, being reposted by around 22,000 people. Each of these reposts had the links to the image deleted. After many women complained to *Facebook* about the policy and flooded the site with their own breastfeeding selfies, *Facebook* eventually reinstated the image, claiming it was taken down in error [15, 16]. Although *Facebook* now claims that breastfeeding images are welcome on the site, it maintains that it must respond to any reports of indecency or nudity (often phrased as being in the interests of children) and as such there is a caveat to which breastfeeding images are deemed appropriate. As a result, thousands of women around the world have had their breastfeeding selfies removed or blocked by *Facebook* after being deemed to be in breach of the site’s ‘Community Standards’ for decency. A similar incident had occurred several years earlier, in 2008, in North America which resulted in on- and offline protests. At the time, a spokesman for *Facebook* explained ‘Photos containing a fully exposed breast, as defined by showing the nipple or areola, do violate those terms on obscene, pornographic or sexually explicit material and may be removed.’ So, images of breastfeeding are not problematic per se, as long as a nipple cannot be seen [17]. The nipple and / or areola automatically codifies the breast as a sexual breast, and it is seemingly impossible for the breast to be signified as sexual and desiring *and* nurturing and feeding at the same time [18]. Just as women who breastfeed in public are expected to adhere to the unwritten rule of ‘discretion’ whilst feeding (read: no exposure of nipple and / or areola), the online depiction of breastfeeding is subject to the same disciplinary regulation. Discretion is mandatory and nipples should not be visible under any circumstances!

## The case study

This paper works with a detailed case study taken from UK based research on a larger project considering the psychosocial significance of the phenomenon of breastfeeding selfies. All names used hereafter are pseudonyms to protect the participant’s anonymity. The focus of this case study is Yasmin, who has shared her own breastfeeding selfies online and who also regularly participates in online breastfeeding and mothers’ groups. Yasmin is a 32-year-old single mother of two children, Summer aged 7 and Adele aged 2. Adele continued to be breastfed, whereas Summer had been breastfed and bottle fed with artificial milk concurrently until she was seven months of age, after which time breastfeeding stopped and Summer continued with artificial milk only. Yasmin was no longer in a relationship with either of her children’s fathers, although Adele’s father still very much played a significant part in both Yasmin’s and the children’s life. Yasmin had been recruited from a ‘closed’ online breastfeeding group on *Facebook* and was one of 12 respondents. This particular online group was selected because it was started in the UK, the focus of the larger research project, and is specifically dedicated to breastfeeding older babies and children, understood as being over 12 months old. This is of particular importance to the researcher, not just because of her own experience of breastfeeding a three-and-a-half-year-old and the stigma attached to this, but also because it is frequently the age of the breastfed child that becomes the focus for the contentiousness of breastfeeding in the cultural arena.

Yasmin was interviewed in depth over two sessions, one week apart, in a café near to where she lived. Adele was with her for both interviews and there was a 30-min break during the second interview to allow Yasmin to take her to nursery. The researcher drew upon phenomenological [19, 20] and psychoanalytically inflected research methods [21, 22] for narrative analysis in both the collection and analysis of the data. This approach was considered most suitable given the centrality of subjectivity including the dynamic of interviewer and interviewee in co-creating the interview, and reflexivity in these research methodologies. Additionally, it was considered imperative that techniques employed to analyse the data allowed for a nuanced consideration of both the internal processes and external forces that inform each other in the practice of breastfeeding. The first initial interview followed the format of asking three broad, open-ended questions with the specific aim of inducing narrative: 1) Can you tell me your life story? 2) Can you tell me about your breastfeeding journeys? and 3) Can you tell me about your experience of taking and sharing breastfeeding selfies? During the second interview, Yasmin was asked specific questions, unique to her story, according to what she had discussed in the first interview. Nothing was introduced that she hadn’t already mentioned in the first interview, and wherever possible, the same language, words and phrases were used in devising the question. The questions were all open-ended, following the same format: ‘You told me about “.. .” , can you say a little bit more about this?’ Questions for the second interview were devised in relation to a consideration of the transcript content from the first interview. The transcript was coded according to emerging themes, and then into sub-themes. In addition to thematic questions, the researcher also noted any examples of ambiguity; contradiction; gaps, silences or a drifting off; changes in tonality or emotionality; and shifts or associative connections, and questions were devised in relation to these speech acts. This two-pronged approach in formulating follow-up questions enabled a move towards a psychosocial understanding of the lived experience of each participant, in both the sense of their life as a social subject and also the inner workings of their psychic life. Hollway and Jefferson [22] draw attention to the fact that we are all ‘defended subjects’ and it is moments, such as in the examples of those given above, that need to be considered carefully if we wish to consider some of the dynamics of unconscious processes. The interrelation of both inner and outer worlds has been held on to when considering the significance of ‘meaning-making’ in the context of breastfeeding. This follows a psychoanalytically informed notion that ‘we are inhabited by our histories of past relationships, and that past experiences, our own and those of others, structure our inner experience and relational possibilities in the present’ [23].

Out of the existing participants, Yasmin’s story was chosen for this particular article because, on paper, Yasmin would demographically fall into the category of mothers considered to have the lowest initiation and duration of breastfeeding in the UK [24]. Statistics suggest it is young, white, working class women who have the lowest breastfeeding rates in the UK [25]. Yasmin described her ethnicity as being ‘mixed’, both white British and Turkish. She left school at 16, having been educated to GCSE standard. She was not currently in paid employment and was in receipt of state benefits. She had recently started undertaking some voluntary work in the same sector as her previous employ (shop work). Yasmin’s case highlights how the relational practice of selfie taking and participation in the heterotopic spaces of online breastfeeding and mothers’ groups helps develop an embodied sense of symbolic capital which has ramifications in the everyday, although not without its own contradictions. I suggest that a focus on the relational, communicative dimension of breastfeeding selfies taking place within online spaces, gives valuable insight into what is being communicated *psychosocially* between women. By holding on to the specificity of one individual, I highlight the nuances and complexities of the practice of both sharing and consuming breastfeeding selfies, and tease out how the practice contributes, in the specificity of Yasmin’s case, to a taking up of a maternal subject position. Attention is paid to the ways in which the practice of taking and sharing breastfeeding selfies is both informed by and informs one’s own inner reality as well as is inextricably bound up with one’s own external social positioning and materiality.

## Discussion

### Symbolic capital of breastfeeding

The position that social discourses around breastfeeding have in relation to larger cultural discourses on motherhood, means that breastfeeding carries a certain symbolic capital, a capital which is intimately connected to the capital of ‘good mothering’. Symbolic capital refers to an attribute or practice that is legitimated or recognised through the ways in which it is valued within a culture [26]. However, the symbolic capital of breastfeeding in the cultural discourses of motherhood, is not without its contradictions, and breastfeeding is positioned as the most superior method of infant feeding, yet women are still shamed for breastfeeding in public and support for breastfeeding women is still woefully lacking [18].

Yasmin told me about a friend who suffered online abuse due to her breastfeeding an older child after she’d shared a post about this on her own social media:

“myself and one other friend stuck up for our friend, you know, ‘well actually it’s very natural, this that and the other why do you think it’s like that?’ And I’m saying to her, I’d like to know your honest opinion I’m not, you know, I’m not saying what I’m saying in a judgemental tone so don’t take it like that der der der der der. .. and. .. she couldn’t answer the questions. And in the end, erm, this friend, alth-, she is normally very, very shy, and in the end, she just deleted and blocked this other girl. .. but I’ve, I messaged the friend and I was like, ‘look I’m so sorry that it escalated out of control but she was being disgusting.’ She went ‘no, d’you know what? I’m actually so thankful that I had you and at the seco-, you know, another person, so, you know, like three people actually sticking up for me and my choices’. And it’s like well it’s not just sticking up for you, it’s sticking up for breastfeeding.”

The internalisation of the potential for negative reactions toward breastfeeding an older baby is, unfortunately, a common dimension of maternal experience in contemporary Britain. Breastfeeding mothers are able to draw upon a shared knowledge of the symbolic capital afforded to breastfeeding in order to defend against such reactions. Furthermore, breastfeeding not only signifies a form of symbolic capital in the context of mothering, but also acts as a signifier to a larger community of (breastfeeding) mothers who are in alliance, whether one knows many breastfeeding mothers in ‘real’ life or not.

Since being named the ‘UK’s number one parenting trend’ in 2015, breastfeeding selfies have continued to maintain a presence on social media in the UK, indeed they can now be seen as a very ordinary representation of motherhood in the digital age. Many women share these images with ‘friends’ or ‘followers’ on their own social media accounts, as well as frequently sharing these images amongst communities of breastfeeding women via online groups and forums dedicated to the experience of breastfeeding. As every technology brings new forms of social and emotional entanglements [27], breastfeeding selfies can be seen to function as a particular relational encounter between the women who produce and consume these images. As such, it is not just the individual mother and her image which provide an articulation of maternal subjectivity, but it is also the intersection of those who consume and interact with the image which contribute to this articulation, and this in turn has a relational impact on the surety with which an individual takes up a maternal subject position.

### Internalised ambivalences

Nonetheless, despite the symbolic capital given to breastfeeding, and also the potential for this capital to flourish in online spaces, the continuous internalisation of the possibility for negative reactions means that breastfeeding women need to have continuous defence mechanisms in operation. These defences need to be more rigidly in place for women whose positionality means that breastfeeding is not the norm, for example in this case working class women, and this can encourage an even more acute ‘splitting’ as part of these defence mechanisms. This internal conflict is a symptom of the wider cultural ambivalence toward breastfeeding. Perhaps the act of taking a breastfeeding selfie is in part motivated by the unconscious desire for a sense of cohesiveness, where the singular image helps foster a sense of completeness or wholeness, defending against the possibility of fragmentation or fracturing of a sense of self. A very common example of splitting resulting from the cultural ambivalence toward breastfeeding, is the difficulty of the maternal and sexual breast to coexist. It is almost entirely impossible in current cultural imaginary for the maternal *and* the sexual to coexist in the *same* breast, at the *same* time. Generally, it is only within feminist writings and artistic practices that the mutuality of the two is not only recognised but celebrated [8, 28–30]. Outside of this domain, at the level of mainstream culture, it is an impasse in the cultural imagination leading to a woeful lack of adequate cultural representations and thus resources, for breastfeeding mothers. Groleau et al. research into young white working class women in Canada shows how the symbolic capital available to these women is located in their desirability, as such the breast must stay as the desired, sexual breast [31]. Groleau suggests that this is a major contributory factor in explaining why breastfeeding rates are low within this group of women: ‘they feared that on top of the toll that pregnancy had already taken on their bodies, breastfeeding would deform their breasts, the symbol of their attractiveness’. These young women lack many forms of capital, except the symbolic capital accrued from performing as a ‘good mother’ within particular social fields and capital acquired from their desirability. It is this latter form of symbolic capital which Groleau argues is the most influential in these young women’s feeding choices [31]. For Yasmin, it was the symbolic capital associated with ‘good mothering’ that was most influential, the cultivation of which necessarily required a distancing from the sexual potential of her breasts, so that the highly valued maternal function remained uncompromised:

“It was a case of. .. for me, that is what they’re [breasts are] for. .. so, yeah, I was very determined to breastfeed”.

“I have always been one of these. .. you know, to not go near it. .. you know, sort of like with partners, it’s like you don’t need to go near it, they’re not for *men*. ..”

The imagined inability for the both the maternal and sexual breast to coexist in the cultural imaginary, was very much internalised by Yasmin, her breasts had a maternal, not a sexual function. When I asked her whether she had this attitude prior to having children, she said:

“Pre-children, yeah, erm. .. for some reason it just never. .. it was like, ‘what are you doing? You’re not a baby!’. .. I mean you don-. .. yeah. .. It’s one of these, I would, I sort of like have to move away or wear a top.”

The fact that she refers to her breasts as ‘they’ or ‘it’ further emphasises this split, not only at the level of the maternal and erotic, but also as a fracture within her own maternal embodiment. A factor that greatly influenced her aversion to dry nursing or nursing for comfort not food:

“if she then stops sucking it’s then like right, ok, you were just clearly comfort sucking for. .. no general rhyme or reason, you know, sort of like you weren’t getting any milk out, so off you pop, you know, back into your cot, or into your bed, or whatever.”

“I just find it. .. strange and. .. yeah, sort of, there’s. .. not a need. .. for it, you know from a personal view, so it’s like, no, you can get off me now.”

And yet Yasmin’s awareness of the symbolic capital given to breastfeeding in the context of ‘good mothering’ means that she can perceive others’ breastfeeding practices different to her own. When I asked her about how she feels about her friend who was breastfeeding a four-year-old, she said:

“I just think ‘aww’. You know, sort of, they’re. .. you know, they’re, it’s amazing that you’re still doing it. You know, absolutely fantastic. I don’t know if I’ll still be doing it at that age. .. but, yeah, I just think that is brilliant, you know, that. .. you’re still able to do that for your child that, erm,. .. it’s not even a case of oh, that they should have dried up and be done, you know, be done with doing that but it’s just the sort of. .. amazing that, you know, child is still getting the comfort and nutrition and everything else. .. from breastfeeding and that mum is still more than happy to do it. But, yeah, I just, I just think it’s fantastic with other people, but, yeah, in my mind, it’s like can I see myself doing that, at that age?”

She was not able to tolerate the notion of the breast as comforter in her own breastfeeding experience due to internalising the split between the maternal and sexual breast but was able to value it when depicted by others. Yet knowing the cultural value given to the breast as comforter, in relation to understandings of a baby’s psychological wellbeing, she could not always admit that she struggled with this intimate dimension of breastfeeding. As can be seen when I asked her about another mother at her daughter’s school who had praised Yasmin for her commitment to breastfeeding, she said:

“I am proud of myself that it was just me that kept her alive, you know, not only sort of the 9 months before she was born, but all this time after she was born til I, til we chose to wean at, you know, around the 6-month mark and it’s still me that. .. is. .. sacrificing. .. to keep her going, to give y-, her that extra nutrition and the comfort”.

Breastfeeding is given value in discourses of good mothering in relation to both its nutritional value and its ability to comfort, to soothe, to communicate. Yasmin is aware of this, and wants to be able to relish in all of these dimensions, but is unable to delight in the physical sensations of breastfeeding. Part of this is the conflict between the ways in which breastfeeding is represented in the cultural imaginary: the imagined impossibility of the maternal and sexual being able to coexist in mutuality [32, 33], and also the presentation of breastfeeding as ‘natural’ and intuitive rather than a learned, cultural practice. A breastfeeding selfie has the potential to gesture towards all the experiential possibilities of breastfeeding – the nutritional and functional as well as the intimate and sensual dimensions. For those consuming these images polysemic interpretations are of course possible, if not likely, and this potential enables the mother taking the photo to signify all dimensions of breastfeeding, without necessarily experiencing this sense of cohesion herself.

### Perceptions of offline support

Yasmin’s perception of the extent of paternal support also greatly influenced her experience of breastfeeding. Comparing her initial breastfeeding experience with Summer, to that of Adele, paternal support (or her perception of that) was a crucial factor in her breastfeeding success:

“especially having someone like [Adele’s] dad being so supportive as well. Although some people would sort of, if they, if they had heard some of the things he said they’d be like that’s a bit out of order. Erm, oh he, erm, he’d just be like ‘oh just man up, you know you can do this, this is what, you know, this is what they’re for, just do it, stop your crying’, and he wouldn’t even say it in like a cross voice, it’d just be like. .. a matter of fact. He’s a very matter of fact person.”

In this context, breastfeeding ‘support’ does not involve emotional support, such as empathy or sympathy for some of the difficulties that breastfeeding mothers can experience. Instead, it is entirely rooted in the maternal *function*. As long as Adele’s father is not telling her to stop breastfeeding, this feels like support to Yasmin.

“He’s even said, I’m not anti-formula, I’m just pro breast. Those, those were his words. . .and it was like, erm, I don’t want to say that I’m anti-formula but. .. it. .. because it does have its place but. .. no breastfeeding or we find another way.”

Adele’s father’s position as being ‘pro-breast’ and believing that ‘this is what they’re [breasts are] for’, is exactly the same language that Yasmin uses to describe her own position on breastfeeding and her perceived purpose of the breast. As such, there is a blurring of their two positions, is one of them mirroring the other? And if so, what are the power dynamics at play in the process of influence?

It was important for Yasmin’s sense of agency to present herself as assured and autonomous when it came to issues around mothering:

“Yeah, erm. .. I would say in terms of like, erm, parenting that I. .. erm. .. I wouldn’t let anyone. .. walk over me or try and pressure me into doing things that I didn’t want to do.”

An influential factor in her need to present as a self-assured mother, was due to the difficult relationship she’d had with her first daughter’s father. She described herself as ‘a naïve mum’ first time round, referring to both her lack of assertiveness within this relationship and also in her interactions with health professionals at the time of Summer’s birth. It is through practical experience and the accrual of knowledge gleaned from online resources that she has been able to negate this sense of being ‘naïve’, and with this comes a more assured sense of her maternal subject position. Breastfeeding selfies have played a part in this development, as can be seen from the difference in her engagement with digital media with both children. On the subject of capturing images of breastfeeding her first daughter, she said:

“I just kind of look back and, and sometimes think that. .. you know, although I’m. .. glad you’re [Summer’s] here, I wish the circumstances were a lot, a lot different. .. And. .. Yeah. You know, sort of like, as she got older there were pictures but, yeah, I’m, I’m 99 % certain there wasn’t any sort of early, *early* months’ ones”.

Images can trigger painful memories, and the lack of images of Summer in her early life is in part due to the emotional difficulties that Yasmin was experiencing due to a continual lack of support and devaluation. Being able to look at oneself from the outside, from another vantage point, has a particular function in the development of a maternal subject position. Thus, the function of ‘mirroring’ Adele’s father’s position on breastfeeding (or him mirroring hers), serves a similar purpose when it comes to gaining a sense of a secure subject position. A commitment to breastfeeding becomes legitimated when perceptions of its value are reflected back, although this is of course not without its difficulties when only certain aspects e.g. the functional, nutritional dimensions, are valued.

Existing research clearly indicates that whether a mother feels supported or not in her decision to breastfeed is a crucial factor influencing both the initiation and duration of breastfeeding [34, 35]. Research also suggests that negative reactions to breastfeeding can greatly influence a woman’s decision-making in whether she attempts to and / or continues to breastfeed [36, 37]. Furthermore, such reactions need not only be experienced at the micro level of personal experience, but also impact upon the breastfeeding mother through their circulation in wider cultural discourses. Whilst steps may have been taken to institutionalise breastfeeding support, through schemes such as the Baby Friendly Initiative and legislation such as the 2010 Equality Act, breastfeeding mothers still need to negotiate the cultural ambivalence to the practice at both a micro level (e.g. in the context of familial or social networks) and a macro level (e.g. breastfeeding in the social, public arena outside of specifically designated breastfeeding spaces). The sharing of breastfeeding selfies and the positive reception of these images, particularly in online spaces, helps facilitate a sense of confidence in many women, although this does not *necessarily* always lead to increased confidence in breastfeeding in public offline.

### Lack of cultural resources

The difficulties of birth and early motherhood for new mothers cannot be adequately prepared for due to a lack of symbolic support or available cultural resources on the realities of birth and breastfeeding.

“It was like, mmm, surely this is not how it’s supposed to be, you know. This is not how. .. you know, you’ve, I’ve seen pictures of it, or you know, this is not how I imagined, *definitely* not how I imagined it. I imagined the cradling of this little bundle. .. and. .. yeah, just. .. just. .. having, yeah, that sort of closeness and you definitely don’t feel like, it’s like a hoover being on you. Being stuck on you at full whack.”

Yasmin was not prepared for the physical sensation of breastfeeding. The existing imagery fostered an idea of an experience without sensation and Yasmin had not been exposed to conversations around the ways in which breastfeeding might *feel*. The dichotomy of the sexual and maternal translates into the split between feeling and not feeling, or experiencing sensations and being sensationless. As well as selfies, short breastfeeding video clips are also commonly shared in online breastfeeding groups, and each post opens up the space for discussion and interaction. Homemade short breastfeeding videos tend to be humorous, depicting children making loud or strange sucking noises, or talking, or moving around in all sorts of contorted positions whilst breastfeeding for example. Both the selfies and videos contribute to the burgeoning repertoire of cultural resources that are potentially available to mothers [8]. Online breastfeeding groups occur at the intersection of alterity (i.e. at the point of separation from the offline world) and difference (i.e. where being a breastfeeding woman is the ‘norm’) and it is within this location that such representations can flourish. This seems possible because there is more potential for knowledge, power and influence to come from the ‘bottom up’, from mothers themselves although of course these channels are not immune from a different kind of regulation or disciplining. Nonetheless, it is within these online spaces that collectively women are the ‘experts’ and in the position of knowledge, and with this shift comes a greater acceptance of the heterogeneity of the depiction of breastfeeding. Perhaps if there were a greater variety of representations in the cultural arena, then women wouldn’t feel so surprised at the variety of potential breastfeeding scenarios and breastfeeding would be anticipated as an active, not a passive process. The internalisation of cultural binaries such as the activity of breastfeeding compared to the passivity of its representations contributes to the fracturing of the cohesion with which women can experience their own corporeality of breastfeeding.

The lack of heterogeneity in available cultural resources on breastfeeding, and an absence of transparent dialogue on the subject, sets mothers up to feel inadequate when they find themselves struggling with breastfeeding, this supposedly ‘natural’ intuitive practice. The fantasy that Yasmin had of the ‘naturalness’ of breastfeeding came crashing down after the birth of her first child. In the trauma of the birth scenario (an emergency Caesarean section), Yasmin experienced an acute sense of a loss of agency and an inability to smoothly adopt a maternal subject position.

“I was telling the nurses. .. you know, I need help with feeding, I need help with feeding. Five times it took for someone to actually pay attention to me. She hadn’t been fed at all from, erm, for the first six hours of life. She hadn’t had anything. .. and I was eventually, erm, given Summer. My boob grabbed and grabbed and pulled about and. .. then literally baby sort of pushed up against my chest.. .ok, so this is a bit strange”.

She goes on to say:

“eventually they said, you know, we need to give her something, we need to give her formula. No, just let me breastfeed, just let me breastfeed. I eventually relented and said that she could have formula but this is only because I was doped up on sleeping tablets because everyone else on the ward has their baby with them and they’re crying. I didn’t have my baby with me so I asked them for something just to help me sleep, help me drown out all the noise around me.”

Without reassurance or guidance, mothers can feel guilty and lacking:

“I was definitely one of these naïve first time mums, that I thought I was going to have the smooth labour, that if, that if I was offered pain relief, I’d take the pain relief. That, you know, my body was gonna do. .. what it should do and. .. yeah, I do feel like my body failed me {ST: do you?} Yeah. And I do, I do resent that. ..”

And this guilt is constantly assuaged through how Yasmin internalises both the fantasy and reality of breastfeeding. It shows the tension between knowing the cultural value ascribed to breastfeeding, and wanting to incorporate this, yet being unable to do so for a whole host of psychic and social realities and histories. This feeling of resentment leads to further fractures in the sense of an embodied maternal subjectivity, further aggravating splits. Breastfeeding selfies have helped Yasmin go some way to healing this split and reintegrating her maternity into a sense of a cohesive embodiment, as became clear when I asked her how it feels to look at these images again:

“I think it’s great because it’s like. .. look at, especially sort of, look at the. .. how. .. look at how small you were. .. and especially for. .. before weaning age. .. *I* did that. *Everything* that you have become, you know, all the little rolls, all you know, sort of like everything that was sort of growing, it was. .. I’ve done that. .. no one else. ..”

### Acknowledgement and achievement

When Yasmin spoke to me about the number of breastfeeding selfies she had taken, she had told me that she had taken more breastfeeding selfies than she had posted online. And the ones she had shared tended to highlight “breastfeeding milestones”, such as the first feed or after one year. The notion of a ‘milestone’ corresponds with linear, cultural time, which points towards the need for acknowledgement at the wider level of social structure. Yasmin believed that she had uploaded an image she took when Adele had her first breastfeed as a two-year-old, but when looking at her *Facebook* uploads when we were together, she realised she had not actually posted this image:

“I, I *thought* that I had posted a picture of her, erm, at 2 but I’m guessing. .. that I. .. didn’t and that it’s only one of these on my phone. .. erm. .. which. .. little bit disappointed in because, yeah, I do normally, proudly. .. you know stick them on there and, erm, and it’s one of these don’t overly. .. don’t overly care. ..”

These contradictions or blurring of memory are interesting for different reasons. On the one hand, it shows how the recording of the breastfeeding journey by taking (and often sharing) images forms part of the embodied memory of the mother, with all the inconsistencies of any memory, but also that this example shows the ways in which Yasmin has internalised some of the stigma attached to breastfeeding older children (i.e. beyond 12 months old) and despite her confidence and surety, has not actually been sharing any breastfeeding images beyond the first year. There is a conflict between one’s lived reality offline and the position one can take up online [8].

Yasmin also showed me a collage she had made to celebrate reaching one year of breastfeeding. It consisted of a series of images showing Adele’s physical growth and development over the course of the first year, with some images of her breastfeeding, and in the centre of the collage was a text box stating: ‘breastfed for one year’. Commenting on this image, she said:

“I felt that I needed to put, erm, the ‘breastfed for one year’ right in the middle as that was sort of like literally my badge of honour, sort of like my badge of achievement”.

Yasmin clearly felt a sense of pride in her commitment to continuing breastfeeding, a sense that provoked some discomfort in her:

“I probably did make a fair few braggy posts on *Facebook* about that [being proud of one’s breastfeeding achievements]. . .erm.. .becau.. . although some people would see it as braggy but it’s just like nah.. .”

The difficulty Yasmin felt in admitting a sense of pride is linked in many ways to her social positioning. As a single mother of two children with two different fathers, unemployed and in receipt of state benefits, the ‘type’ of mother she potentially signifies is one who is vilified and abject in cultural discourses [38]. There is therefore a sense of conflict in whether she feels she has the right to celebrate her achievements and articulate her pride. This is indicative of the social structure which removes mothers of their specificity and glosses over the impact of social and material pressures. Yasmin’s sense of pride contributes to her self-esteem, thus assists in the surety of her social position of being a mother and is thus in conflict with the ideological imperative to either idealise or denigrate mothers. Yasmin’s pride does not disavow the pain, suffering and difficulty of breastfeeding, instead these oft-ignored dimensions of breastfeeding are precisely what contributes to her sense of pride in the first place. This is a radical disruption to many other cultural images available to mothers.

Yasmin showed me one image she had posted on her personal *Facebook* page, it was a comparison image with a photograph of Adele at one-hour old breastfeeding on the left-hand side, and then at a year-old on the right-hand side. The caption read:

“less than an hour old and one year old shocked myself what my body can do for my baby bloody exhausting but worth it.. .”

The ability to see an image of one’s own breastfeeding journey assists in an external realisation of an embodied cohesion, a positive connection which is essential to emotional well-being. A sense of achievement, thus pride, is aroused in the mother, one which demands, or at the very least is a call for, a larger social recognition of the physical and emotional labour of breastfeeding.

Yasmin spoke to me about the reaction she had to posting this image on her own personal *Facebook* page:

“Do you know what, I was actually quite gutted because I didn’t get much of a response at all.. .”

She went on to say:

“I was expecting *something*. .. I would say I maybe had about. .. sort of 10 likes. ..”

The symbolic capital associated with breastfeeding is of particular value in online spaces dedicated to breastfeeding, and often in spaces dedicated to mothering more generally. This capital does not seamlessly traverse into the offline ‘real’ world, neither does it necessarily carry the same value in wider online spaces that do not have mothering or breastfeeding as the common reference point. Of course there is also a counter to this, in the form of the ‘Fed is Best’ campaign for example, in online spaces dedicated to mothering. The physical and emotional demands of breastfeeding, and mothering more generally, were sometimes painfully felt by Yasmin, but without the space to articulate these difficulties, what she sought was recognition of these difficulties. The ability to share breastfeeding selfies was a gesture towards insisting on this recognition, an open address to the other to acknowledge the reality of breastfeeding. Recognition became a key theme throughout our interviews, and seemed to be a guiding influence in Yasmin’s on- and offline breastfeeding experiences.

### Importance of recognition

Breastfeeding selfies are portrayals that not only the mother who took the image can look back on, but also can potentially be seen, acknowledged, and recognised, by others if the image is posted or shared online. The notion of recognition here is two-fold: it involves an acknowledgement in the philosophical sense ‘as a process or action, the essence of responsiveness in interaction’, for example the recognition of Yasmin’s achievements; and also, it refers to an intersubjective and intrapsychic process of recognition in which ‘we know the other’s mind as an equal source of intention and agency, affecting and being affected’ [39]. Yasmin had been breastfed as a child, and had a certain amount of pleasure knowing that she had in fact been breastfed for longer than her younger sister.

“I was breastfed until between sort of 2, 2 and a half as well, erm.. . I was actually breastfed even after my sister was born, err. .. erm there’s 17 months’ difference. I was still breastfed even after my sister was put on formula. .. so yeah. .. even though, even though I’m the older child. My sister, yeah, my sister was put on SMA and then, erm, yeah, I was still being breastfed, I was still breastfed for a couple of months apparently. .. erm. .. which yeah was quite surprising. ..”

If we consider the notion of recognition as a continual oscillation between ‘relating to the outside other and the inner object’ [39], it could perhaps be suggested that it was her own experience of being breastfed which cemented Yasmin’s ideas that breastfeeding needed to be a central component of her maternal subjectivity. As it was in this context, that breastfeeding seemed to function as a gesture toward recognition, a recognition which was generally felt to be lacking in the familial structure:

“I also found that all his [her father’s] other children look exactly like him. So, I just felt that they got special treatment for that.”

and social structure:

“Erm. .. I, because I was known as the shy one, I was always known as ‘oh you’re blah, blah, blah’s sister’, ‘you’re blah, blah, blah’s sister’, ‘you’re blah, blah, blah’s daughter’. You know, sort of, I was rarely known by my name. and. .. yeah, that was frustrating so it did get to a point where I would, I wouldn’t speak to people unless they knew my name.”

Yasmin’s acute need for recognition, relating back to her childhood, meant that seeking recognition was sometimes more crucial than ensuring good practice. In the first interview, Yasmin had described the experience of breastfeeding her second child for the first time:

“she was an elective C-section and. .. erm. .. I’d already let the hospital know that I’m going to be breastfeeding, ‘ok fantastic, right we can sit you up, and sit you up, lovely, how does that.. . how’s that feel? are you comfortable? here’s your baby’, and she latched on like a pro. .. It was like *yes*! *This* is it! and. .. then. .. they came in about an hour and half later while I was in recovery, to check you know how the anaesthetic’s wearing off. .. ‘Oh how did she breastfeed?’ I was like ‘oh she had this side for this length of time, this side for this length of time’. ‘Fantast. .. ’ and they was [*sic*] so enthusiastic and it was like wow!”

Shortly after this, she said:

“yeah she was latched wrong for the first 24 hours. .. and I knew she was latched wrong, I just wanted to get feeding done and then for. .. erm, me to get the half hour’s sleep I was allowed until the next feed. .. so it got to the point of, erm,. .. of, erm, cracked, bleeding nipples.”

This example shows that recognition (in this case from health professionals) was of key importance to Yasmin. She needed to be seen to be doing the right thing. In many ways, this links back to her trying to work through the trauma of the birth of her first child, when she felt her body failed her. Yasmin believing that she was doing a good job (or in this case, acknowledging that the baby was not latched properly) is not enough, the actions need to be recognised from the outside. The crucial factor for Yasmin, was that her efforts and ability were recognised, that she was recognised and respected as a ‘good mother’. She was willing to endure pain and discomfort to gain the recognition she so desperately craved.

After the birth of her first child, Summer, Yasmin felt as though her wishes were not being respected, her voice not heard, and effectively she experienced an erasure of subjectivity. Her position was usurped by both health professionals and her then partner. *She* was not being recognised.

“erm, yeah, sort of, like I think round about the 8-10 week mark, health visitor, oh she’s dropping weight, oh, oh, oh, you know this, that and the other, you need to be topping up, you need to be topping up. I’m breastfeeding, I’m breastfeeding. ..”

She goes on to say:

“So yeah we’re erm. .. we’re sort of. .. er. .. not into an argument but it’s one of these, ‘no, I’m the parent this is what we’re going to do’. Eventually she then said, ‘right well what she needs to be weighed twice a week’. .. why twice a week?. .. Well, because the tactics didn’t work on me. .. to. .. erm. .. I’m breastfeeding, I’m breastfeeding, and I wouldn’t give into her. .. she then started talking to Summer’s dad, who I was with at the time. Yeah, ‘Summer’s dropping too much weight, she needs to have formula too, she needs. .. ’ and basically got them both to gang up on me. ..”

Yasmin seemed able to use the ‘success’ of her breastfeeding journey with her second child to negate some of the pain from her experience with her first daughter.

“It felt really, really good because. .. it was like, it was pretty much, yeah, that in a way, that I’d beaten that, even though it was literally the first sort of hours, that it actually felt I’d beaten my demons from having Summer. Because, you know, I just felt that I’d been put down so much over it that it was like, you know, I am gonna do this this time, I’m gonna be successful at it.”

When I asked her how she felt to see some of the images of Adele breastfeeding again, she said:

“Yeah, the things that’s sort of not in the front of your mind. Yeah, it’s just sort of like *throws* itself into the front of your mind and you’re just like ‘aww!’ Yeah, you just, yeah definitely the reminiscing. .. in a positive way”.

For Yasmin, having the space to articulate her experience of breastfeeding and motherhood, being able to express the embodiment of a maternal subject position, is crucial in the extent to which she feels part of a larger social structure, and the taking and sharing of breastfeeding selfies to online publics helps facilitate this.

### Online breastfeeding publics

Online breastfeeding and mothers’ groups can be considered as ‘heterotopias’ [40], spaces which are both related to and separate from the offline spaces they mimic and invert. Within online breastfeeding groups, breastfeeding is of course the norm, it is the common reference point and so a number of the factors regarding the policing of breastfeeding in public spaces in the ‘offline’ world, for example the need for discretion or the expectation that breastfeeding should cease at a particular age, does not apply within these spaces. On the surface, these digital spaces may seem to be utopian, and in many respects, they are, but these spaces are not immune from the functioning of a different kind of regulation. When topics not related to breastfeeding are discussed within these online groups, there is greater potential for a particular kind of policing or regulation to erupt, the question of whether to vaccinate one’s children or not is a particularly good example of such a topic. Non-breastfeeding related topics often emerge through the position that breastfeeding has in relation to a wider discourse of what constitutes ‘good mothering’. Online groups are, therefore, not completely immune from a different sort of policing from within. As such, a particular prevailing discourse around motherhood, and breastfeeding’s central position within it, paradoxically allows the common reception toward breastfeeding to be inverted whilst at the same time exerting a different kind of pressure on the behaviour and practices of mothering.

Nonetheless, online networks and communities of breastfeeding mothers play a key role in offering support and advice to breastfeeding mothers and fostering a sense of safety. Breastfeeding mothers need not feel alone in their endeavour, and thus experiences (positive or negative) can be shared with others. This connection therefore offers some reassurance to mothers who may be experiencing difficulties, be that related to breastfeeding, or more emotionally when one feels isolated or removed from social life due to breastfeeding. As Yasmin said, her phone is her “life line” during long periods of breastfeeding, particularly during the evening:

“I think it helps me pass the time without. .. erm, having to look at the time, because I’ll either pop onto *Facebook* or I’ve got a couple of games on my phone which thankfully hide the time.”

“It’s just nice to. .. erm, I suppose it’s nice to have. .. the escape as well”.

The global reach of digital networks means that communities are available 24 h a day, not just during the normative waking hours of one’s own geographical region. Likewise, the rhythms of breastfeeding, particularly in the early weeks and months, span 24 h with no clear definition of night from day. The nature of digital time allows for a step away from linearity, and a move toward a more cyclical time, which is more in tune with the disregard that early motherhood has toward linear time. The digital landscape has reconfigured our notion of space and time and temporality. [41]

“Yeah, because especially with the smart phones, because you’ve, you’ve got that hand-held support network as well. You know, even if it’s not a breastfeeding group, there’s so many mums’ groups on *Facebook*, or, you know, someone on your friend’s list has either gone through it, or knows someone who has gone through whatever, so can, you know, put you in touch or. .. you know, you can always find something.. .”

For Yasmin, the symbolic capital of breastfeeding becomes usefully applied in allowing her to more confidently take up her own maternal subject position, as can be seen in her role as an administrator on a mothers’ group on *Facebook*. As a currently unemployed, working class mother who was not educated beyond age 16, she has lacked opportunities in the ‘real’ world to be in a position of knowledge or authority. This was something that she clearly craved, as was indicative in the pride she had in telling me that she had worked her way up to stock controller, rather than shop assistant, in her last job. Yasmin’s experience of breastfeeding put her in the position of being an ‘expert’ on breastfeeding in one particular mothers’ group, a position of knowledge and authority that helped to recognise, validate and legitimise her own experience:

“It’s like, you know, I’ve got a use that. .. none of the admins have, you know, I’ve got that sort of. .. yeah, that niche, that sort of, you know sort of like, some, some of them can talk about depression or abusive relationships or. .. you know, lots, you know, lots of chil-, big families, you know, or whatever but for me it’s like my, yeah, mine is. .. breastfeeding. And it’s just like, yeah, I’ll give you whatever advice you want.”

Online spaces dedicated to both mothering and breastfeeding facilitated Yasmin’s surety in taking up a maternal subject position. The capital gained from her breastfeeding experience means that her breastfeeding and parenting experience is valued, which in turn means that she is validated, listened to, heard and has a sense of belonging, that she is part of something. This has been crucial in negotiating the erasure of agency she experienced on becoming a maternal subject when her desires were not listened to or respected, and she was not recognised as a knowing subject.

Online breastfeeding groups invert the logic of the social structure through the centrality of breastfeeding positioned as the common reference point. This inversion removes a significant amount of judgement and stigma around the need for breastfeeding to be discreet, the openness to discuss negative breastfeeding experiences, and what is an acceptable duration of breastfeeding. However, the spaces are not immune from their own regulatory and disciplinary effects, usually around the notion of ‘good mothering’, and this can contribute to a continuation of a fragmented sense of self, whereby mothers are conflicted in what they believe or feel and what they understand as a common expectation of the group. Nonetheless, when we consider that the notion of what is socially acceptable i.e. what is permitted to take place in public, changes over time [18], the impact of the relinquishment of a need for discretion and encouragement of the sociality or collectivity of breastfeeding, which online spaces facilitate, gives some hope to the possibility of a breakthrough or a spilling over of these attitudes into the wider social and cultural arena offline [14].

Engagement with online breastfeeding groups can be hugely beneficial in not only giving advice and offering support through difficult times, but also as a result of the visibility of breastfeeding through the sharing of breastfeeding selfies. The discontinuity between the available cultural representations and the reality of breastfeeding can be negotiated through the acknowledgment, recognition and discussion of the heavy physical and emotional toll that initiating breastfeeding and continuing this practice potentially carries. Failure to acknowledge or recognise the labour required for breastfeeding, and pregnancy, childbirth and motherhood more generally, can be a contributory factor in a mother’s sense of a loss of agency, or sense of the loss of a secure subject position. Stearns observed that women perform breastfeeding in public ‘as though it were a deviant act. .. trying to be discreet and invisible’ [42], and Kate Boyer’s research reinforces this finding in the context of the UK [43]. The imperative for discretion is removed in online breastfeeding groups, and as such breastfeeding becomes repositioned away from discourses of shame and ‘deviancy’. In fact, the contrived nature of selfie sharing insists, or at the very least, *invites* a gaze into a scenario which has been culturally positioned as private and solitary [8]. Fiona Giles astutely observes how the contentiousness around breastfeeding in public, is in part due to breastfeeding being culturally positioned as an individual, solitary act [14]. In other words, it is an act the mother conducts with her child(ren) alone. It is removed from public life, it is not seen, and therefore comes with a notion of assumed discretion. Whereas breastfeeding in public, even if practised individually, has the potential to be seen due to its proximity to ‘others’. It therefore risks transgressing into what is culturally perceived as ‘indiscreet’ – irrespective of how it is carried out. Negotiating ideas of discretion, particularly as linked to cultural understandings of respectability, problematises the possibility for breastfeeding to be reconfigured as a social, communicative act, in other words, as a cultural practice. And it is perhaps for this reason that women seek out online spaces in order to articulate and make sense of their breastfeeding experiences. The sharing of breastfeeding selfies to online breastfeeding groups is a social, relational act. Breastfeeding whilst engaging in social media is a social, relational act. A selfie not only invites a gaze, but also invites comment, be that linguistic (posting on the thread), visual (posting one’s own image in response) or gestured (using emojis). The breastfeeding selfie becomes a communicative tool which not only communicates one particular event (that of the breastfeeding couple) but also more broadly contributes to a communication of the heterogeneity of the breastfeeding experience. Online / offline; public / private; individual / collective; solitary / social, all of these binaries are becoming more blurred in the digital age through our constant interaction and engagement with social media. It is within the murkiness of this blurring of boundaries that a change in the cultural imaginary seems possible.

## Conclusions

Despite the fact that breastfeeding is upheld as the most superior form of infant feeding, there continues to be a cultural ambivalence toward the practice in the UK and breastfeeding rates remain low. This analysis considered what can be learnt from breastfeeding selfies as a relational, communicative practice between mothers in online, digital publics and what this might tell us about some of the barriers to breastfeeding in public in ‘real life’ spaces offline. The case study of Yasmin, referred to in this article, gives a good example of the ways in which these ambivalences may manifest themselves and ultimately be negotiated by mothers who lack symbolic capital. This negotiation is not without its limitations of course and the examples given draw attention to a number of the ambivalences and conflict which ultimately impact upon and shape the lived experience of a breastfeeding mother. For Yasmin, breastfeeding played an integral part in her understandings of a maternal subjectivity and was intimately linked to her sense of maternal agency. Her participation in online groups fostered a confidence in her taking up of a maternal subject position, with ramifications at both a micro- and macro-level, albeit not without their own contradictions and complexities. The practice of taking and sharing breastfeeding selfies functioned in multiply nuanced ways and assisted Yasmin in working towards a more cohesive, coherent sense of self. The lived experience of a breastfeeding mother is inherently shaped and held in tension with prevailing social and cultural discourses, and one’s own intra-psychic relational history. This case study demonstrated the imperative for a recognition and acknowledgement of breastfeeding: in the sense of appreciation of the physical and emotional labour involved; the centrality of the breastfeeding in maternal subjectivity; and breastfeeding as maternal agency. Whilst breastfeeding may take up a particular place in contemporary discourses around parenting and ‘good mothering’, the capital it affords women is inherently wrapped up in positionality and materiality. The result of which is that the need for cultural attitudes to change toward breastfeeding in public will impact some women more than others. As Jessica Benjamin writes, ‘liberation comes not only through *being* recognized but also *doing* the recognizing’ [39]. The impact and influence of the practice of taking and sharing breastfeeding selfies should therefore not be underestimated or trivialised, as it is precisely through their visibility that a relationality emerges, one which could perhaps signal the beginnings of a shift in the possibilities of the cultural imaginary and a move towards breastfeeding being reconfigured as a social practice.

## Data Availability

Data sharing not applicable to this article as no datasets were generated or analysed during the current study. The transcript analysed during the current study is not publicly available due to the nature of the consent gained from the participant and the small study size of the larger project.
